# Rapid diagnosis of Zika virus through saliva and urine by Loop-mediated isothermal amplification (LAMP)

**DOI:** 10.1080/20002297.2018.1510712

**Published:** 2018-09-03

**Authors:** Talita Castro, Maite Sabalza, Cheryl Barber, William Abrams, Antonio Charlys Da Costa, Flavio Augusto De Pádua Milagres, Paulo Henrique Braz-Silva, Daniel Malamud, Marina Gallottini

**Affiliations:** a Stomatology Department, School of Dentistry, University of São Paulo, São Paulo, SP, Brazil; b Department of Basic Science and Craniofacial Biology, College of Dentistry, New York University, New York, NY, USA; c Laboratory of Medical Research, Institute of Tropical Medicine, University of São Paulo, São Paulo, SP, Brazil; d Epidemiological Surveillance and Infectious Diseases, Secretary of Health of Tocantins and Federal University of Tocantins, Palmas, TO, Brazil

**Keywords:** Flavivirus, virology, polymerase chain reaction, RNA, infection, Dengue, saliva, LAMP, Zika

## Abstract

**Background**: Zika virus (ZIKV) is a single-stranded RNA virus and member of the *Flaviviridae* family. Recent studies have reported that saliva can be an important alternative to detect ZIKV. Saliva requires less processing than blood greatly simplifying the assay. Loop-mediated Isothermal Amplification (LAMP) is a rapid assay that detects nucleic acids, including ZIKV RNA.

**Aim**: The aim of this study was to evaluate the efficacy of saliva and urine to diagnose ZIKV infection in subjects during the acute phase, through ZIKV RNA detection by LAMP.

**Method**: A total of 131 samples (68 saliva and 63 urine samples) from 69 subjects in the acute phase of ZIKV infection, and confirmed positive for ZIKV by blood analysis through real time-PCR, were collected and analyzed by Reverse Transcriptase Loop-mediated Isothermal Amplification (RT-LAMP).

**Results**: From the 68 saliva samples, 45 (66.2%) were positive for ZIKV with an average time to positivity (Tp) of 13.5 min, and from the 63 urine samples, 25 (39.7%) were positive with the average Tp of 15.8 min. Saliva detected more samples (*p* = 0.0042) and had faster Tp (p = 0.0176) as compared with urine.

**Conclusion**: Saliva proved to be a feasible alternative to diagnose ZIKV infection during the acute phase by LAMP.

## Introduction

Zika virus (ZIKV) is a single-stranded RNA virus and member of the *Flaviviridae* family [1]. It is transmitted among humans usually by *Aedes* mosquito species, but dissemination by sexual transmission [2], perinatal [3] and blood transfusion [4] have also been reported. Symptoms appear in 20% of infected individuals and include fever, cutaneous rash, headache, conjunctivitis, myalgia and arthralgia [5]. After the USA’s ZIKV outbreak in 2016, a sudden increase in microcephaly and Guillain-Barré syndrome (GBS) related with ZIKV infection prompted the World Health Organization (WHO) to declare a public health emergency of international concern [6,7].

ZIKV RNA disappears from blood in about one week after infection, however the virus remains sequestered up to 29 days in saliva [5] and urine [8]. Since the first report of ZIKV in saliva [9], studies have suggested that saliva could be an important alternative sample to detect ZIKV infection [5,9–12]. Saliva collection is easy, non-invasive, involves safe handling and requires less processing than blood greatly simplifying the assay, and justifying efforts to develop diagnostic techniques through the use of this fluid.

According to Centers for Disease Control and Prevention (CDC) recommendation, the diagnosis of ZIKV infection is based on the analysis of serum and urine by quantitative polymerase chain reaction (qPCR) from symptomatic individuals. Urine collection is also easy and non-invasive, and studies reported that ZIKV RNA can be detected more frequently in urine than in blood during the acute phase [13].

Loop-mediated Isothermal Amplification (LAMP) has high sensitivity and specificity and can detect RNA and DNA without thermal cycling. The assay uses primers designed to target the capsid gene and generate real-time amplification that can be detected in less than 30 min. These characteristics make LAMP assays ideal to use in portable devices, which can be configured to diagnose diseases at point of care facilities [14,15]. Therefore, the detection of ZIKV RNA by LAMP provides the basis of a test that can readily facilitate the diagnosis of new cases.

The aim of this study was to evaluate the efficacy of saliva and urine to diagnose ZIKV infection in subjects during the acute phase, utilizing LAMP to detect ZIKV RNA.

## Materials and methods

This study was approved by the Ethics Committee of the University of São Paulo, School of Dentistry (1.774.973), by the National Ethics Committee for Research (CONEP-Brazil: 1.885.522) and by the New York University Medical Center Institutional Review Board (H10-01894). All participants agreed to participate and signed the written informed consent.

In total, 131 samples (68 from saliva and 63 from urine) were collected and analyzed from 69 Brazilian subjects of all ages that met the inclusion criteria of presenting specific symptoms and signs for ZIKV (acute phase), such as fever, cutaneous rash, headache, conjunctivitis, myalgia and arthralgia; and had confirmed positive for ZIKV and negative for Dengue and Chikungunya virus infection by blood analysis utilizing qPCR, following the criteria described by Lanciotti et al. 2008 [16]. The samples were collected during the acute phase of the disease and the average days between the onset of symptoms and sample collection were 3.2 ± 2.9 days.

Demographic and symptoms information were compiled. Samples of saliva, urine and blood were collected on the same day, in Brazil. Unstimulated saliva was collected with Salivette®, a cotton pad placed in the mouth, under the tongue for a few min and then centrifuged (1,000 rpm/2min) without the use of any buffer. Saliva and urine samples were stored and shipped to USA frozen at −80°C until analysis.

The RNA purification process and the analysis by Reverse Transcriptase Loop-mediated Isothermal Amplification (RT-LAMP) were performed at New York University, College of Dentistry, in a Biosafety Level 2+ facility with appropriate safety procedures, as described in the CDC/NIH Biosafety (BMBL 5^th^ Edition). RNA isolation was performed with a commercial kit (ZR Viral RNA kit. ZYMO® Research) using 300 μL of saliva and 600 μL of urine samples.

Primers targeting a unique sequence in the ZIKV capsid were designed using the Primer Explorer V4 software (Eiken Chemical Co., Tokyo, Japan) with conserved regions of the capsid gene of the PLCal_ZV, Thailand strain (GeneBank accession no. KF993678.1). A set of three primer pairs (Table 1), including two outer primers (forward primer F3 and backward primer B3), two inner primers (forward inner primer FIP and backward inner primer BIP), and two loop primers (forward loop primer LF and backward loop primer LB), were selected. The primers had been purified through HPLC and were assessed for specificity. The concentration was optimized before use in LAMP assays by analysis using the Basic Local Alignment Search Tool (BLAST) of the National Center for Biotechnology Information (NCBI) against sequences in GenBank, indicating that they were ZIKV-specific [17].

**Table 1.** ZIKV capsid primers sequence and optimized concentration.

Primer	5ʹ → 3ʹ nucleotides sequence	Optimized concentration (μM)
F3	GACTTCTGCTGGGTCATG	0.4
B3	GCCAACAATTCCGACACTA	0.4
FIP	CCCCACTGAACCCCATCTATTGGGTCTTGGCGATTCTAGC	2.4
BIP	GTTCAAGAAAGATCTGGCTGCCCCTCGTCTCTTCTTCTCCT	2.4
Loop F	GCTTGATTGCCGTGAATCTC	1.0
Loop B	GCTGAGAATAATCAATGCCAGG	1.0

The ZIKV primers were tested in duplicate for cross-reactivity against Dengue virus (DENV) RNA, serotype 1 (BEI, NR-82 Hawaii) and serotype 2 (BEI, NR-84 New Guinea). Uninfected saliva samples were spiked with tenfold serial dilutions of ZIKV strain (PRVABC59, NR-50,240, BEI Resources) to test the limit of detection of the LAMP reaction.

The RT-LAMP reaction mixture contained a total volume of 25 μL; 15 μL of Master Mix (Optigene®, UK), 0.5 μl of 7.5 units of reverse transcriptase (WarmStart-RT, New England Biolabs Inc.), 2.5 μl of primers (0.4 μM of F3 and B3; 2.4 μM of FIP and BIP; 1 μM of Loop F and Loop B) and 7 μl of template (purified RNA). Samples were kept chilled on ice until placed in the LAMP reaction chamber. The amplification step (30 min at 65°C) was followed by an annealing curve analysis step (98–60°C, ramping at 0.05°C/sec; Genie III, OptiGene, UK). Positive and negative controls were used in all LAMP reactions. Every step of the RT-LAMP assay (time, temperature and concentration of the reagents) had previously been optimized.

Statistical analyses were performed using SPSS® and Bioestat® statistics software. Chi-square, *t*-test, Lilliefors and Wilcoxon tests were performed considering a significance level of 0.05.

## Results

The average age of the 69 participants was 34.7 (± 13.6) years and comprised 46 (66.7%) females. The most common related symptoms were cutaneous rash (56.5%), fever (49.3%), myalgia (49.3%), arthralgia (39.1%) and headache (25%) (Table 2).

**Table 2.** Demographic data.

Characteristics	Participants (*N* = 69)
Average age (range) – yr	34.7 (4 to 75)
Female sex (#,%)	46 (66.7%)
Acute phase symptoms (#,%)	
Cutaneous rash	39 (56.5%)
Fever	34 (49.3%)
Myalgia	34 (49.3%)
Arthralgia	27 (39.1%)
Headache	25 (36.2%)

Samples spiked with DENV with both serotypes did not amplify with ZIKV primers, showing specificity of the ZIKV primers. The limit of detection test demonstrated that the lowest detectable concentration of ZIKV of all replicates (100%) was consistently 2.2 x 10^3^ RNA copies/mL (6.6 RNA copies/reaction). In 75% of the samples, 2.2 x 10^2^ RNA copies/mL (0.66 RNA copies/reaction) were detected.

**Figure 1.**
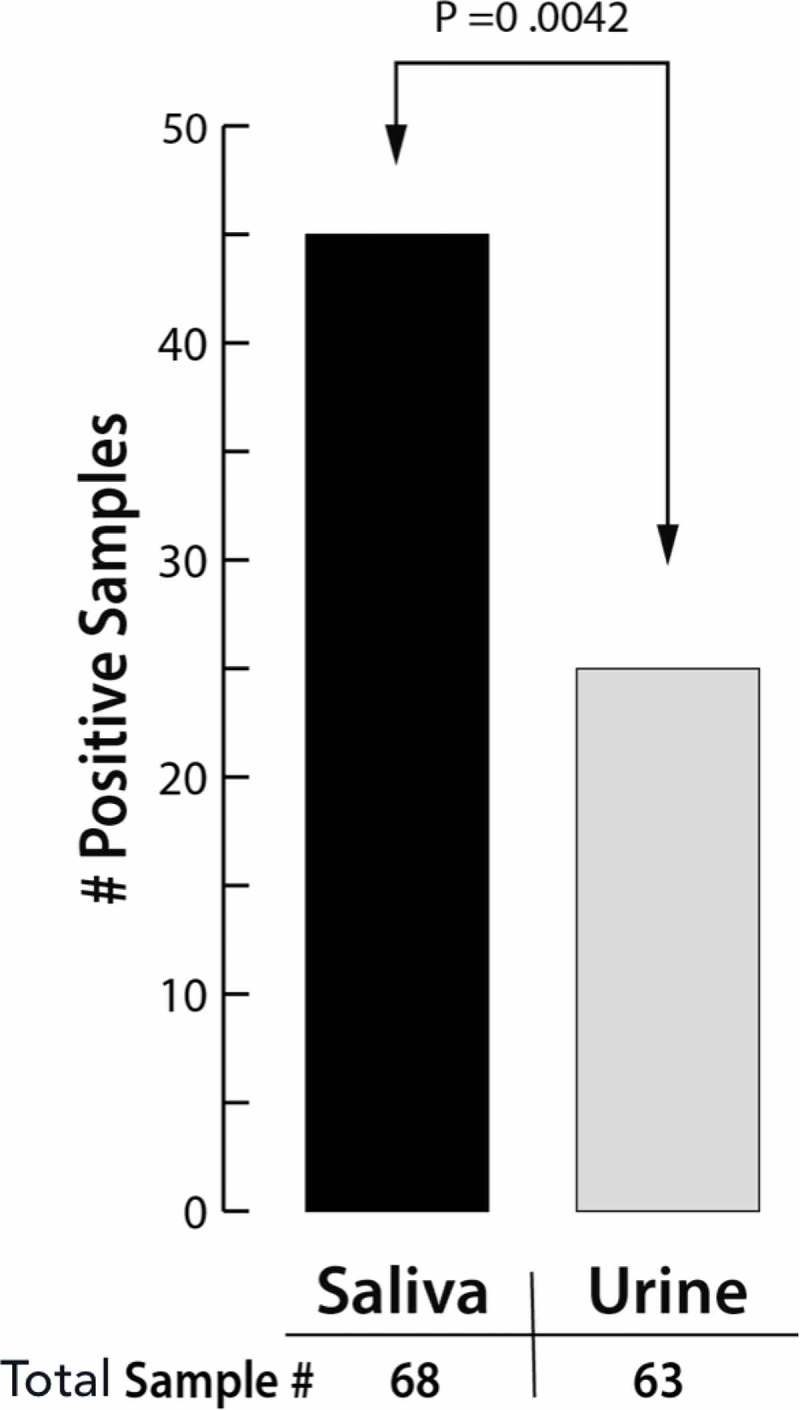
Number of positive samples of saliva and urine and their *p*-value (Chi-square test).

From the 69 individuals, 62 were tested for both saliva and urine. Totally, 21 individuals were positive for both; 23 were positive only for saliva and four were positive only for urine. In total, 45 (66.2%) of the 68 saliva samples analyzed by RT-LAMP were positive for ZIKV, while only 25 (39.7%) of the 63 samples of urine were positive, resulting in a statistically significant difference between them (*p* = 0.0042) (Figure 1).

Additional analysis indicated that the Tp and the detection of ZIKV RNA in saliva and urine did not correlate with age, sex, symptoms or signs and time of sample collection (*p* > 0.05).

**Figure 2.**
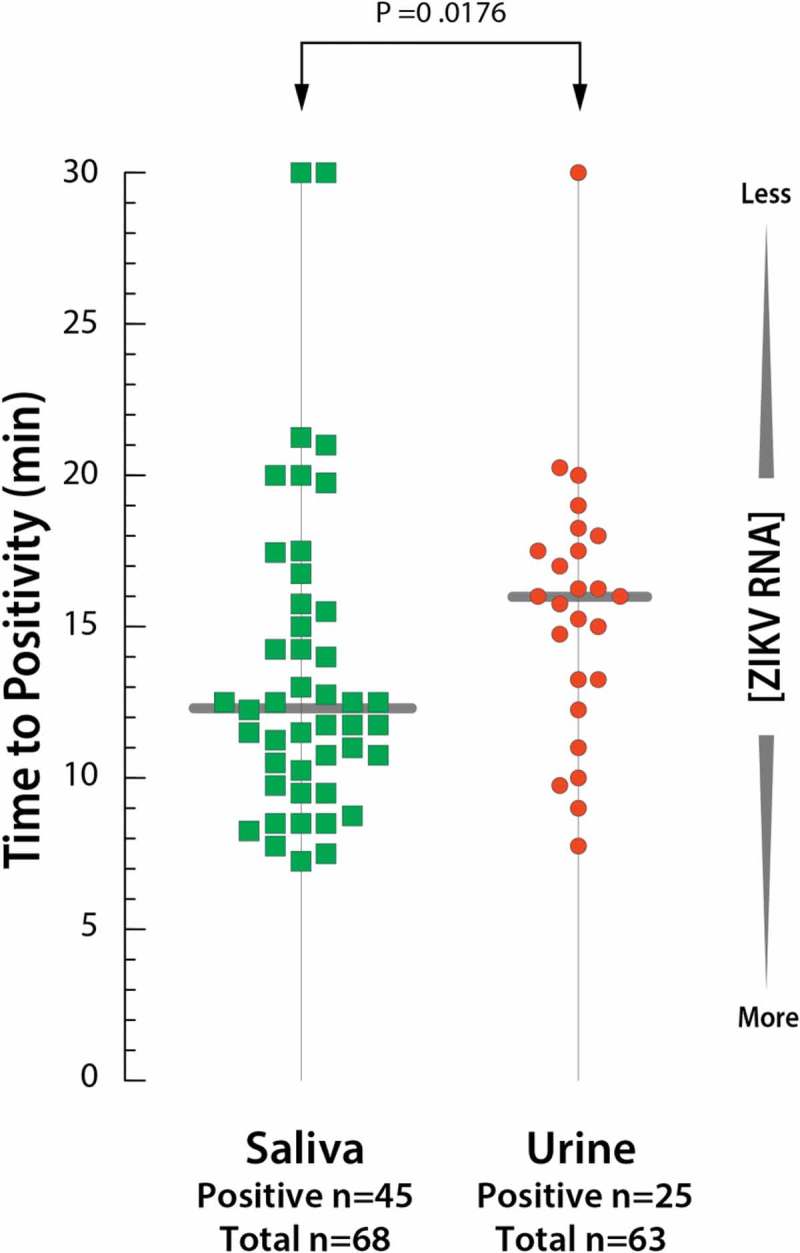
Time to positivity between saliva and urine samples with average and *p*-value (*t*-test).

The average time to positivity (Tp), the time required for the assay to start amplifying the target sequence, was 13.5 min for saliva and 15.8 min for urine, with a statistical difference between them (*p* = 0.0176), even when using half of the sample volume (300 μL for saliva and 600 μL for urine) to purify the RNA (Figure 2). When analyzing the positivity of saliva and urine in the same patient, saliva demonstrated faster Tp in 71.4% (15/21) of the cases.

## Discussion

In 2016, a major outbreak of ZIKV occurred in the USA and consequently a sudden increase of microcephaly and GBS cases related to ZIKV happened. In response, WHO declared an international public health emergency, which necessitated the development of a simple, fast and specific diagnostic method to identify new cases during this and future epidemics.

Saliva collection is a simple procedure, quick and noninvasive for the patient. In this study, saliva was shown to be a feasible alternative sample to diagnose ZIKV infection in subjects during the acute phase using a LAMP assay. Compared with urine, saliva samples detected ZIKV in 1.7 times more cases. Furthermore, the Tp was shorter in saliva and required much less sample volume compared with urine.

Previously published papers demonstrated the efficacy of RT-LAMP compared with RT-PCR to detect ZIKV [18–20]. The LAMP assay has shown to be useful to detect the ZIKV RNA in saliva and urine samples. Nevertheless, most of the previous studies used spiked samples to demonstrate the specificity and sensitivity of the assay. Our study proposed to analyze a significant number of saliva and urine samples from individuals proven positive for ZIKV. The higher number of samples and the fact that real samples were used instead of spiked ones may be the reason why we observed different rates, mainly in urine samples which had a low positive rate in this study. Barzon et al. 2016 observed prolonged shedding of ZIKV in saliva and urine up to 29 days in one patient by qPCR and showed that during the first days of infection, the viral load is constantly changing, and at certain time points saliva showed higher ZIKV RNA (copies/mL) compared with urine [5].

Using specific primers, LAMP demonstrated specificity to ZIKV and showed no cross-reaction with DENV, a common flavivirus in Brazil that usually presents challenges in diagnosis due to cross-reactivity with ZIKV. The fact that LAMP does not require thermal cycling makes the assay simple, fast and cost-effective compared with PCR assay [21,22].

The Genie III (OptiGene, UK) used for the LAMP assay is a self-contained device, which reduces the contamination risk of LAMP reactions. Furthermore, in this study, positive and negative controls were used in all the reactions to assure not having contamination problems. The advantage of observing the results by real-time allows the diagnosis in less than 30 min, so that treatment and care to avoid transmission of the disease can start immediately [23]. Studies already reported ZIKV transmission through sexual [2], perinatal [3] and blood transfusion [4]. The sooner the patient knows about the infection, the sooner the care to avoid transmission could be applied. The characteristics of LAMP associated with the use of saliva samples would be helpful to use in epidemics since it enables the rapid diagnosis of new cases.

Although saliva appears to be a useful sample to diagnose ZIKV infection during the acute phase, other studies have reported a low risk of transmission through this fluid [10,12,24].

This study showed positivity of ZIKV in 66.2% (45 of 68) of the saliva samples. This result implies that ZIKV may not always be excreted through saliva, but at the moment of the samples collection, ZIKV could be detected more frequently than in urine (25 of 63). The major limitation of this study consists in the fact that it was a cross-sectional study, and samples were collected at a single time point. Further studies to better understand the presence of the ZIKV RNA in saliva and urine would require a longitudinal study with a follow-up of the patients and serial collection of samples in different times.

In conclusion, saliva is a feasible alternative to rapid diagnose ZIKV infection by LAMP in individuals during acute phase. The findings of this study contribute to the knowledge of the ZIKV behavior in the organism, which is not totally understood.
